# Supplementary figures and images for: Genome organization: experiments and modeling
Source: Chromosome Res. 2017 Feb 2;25(1):1–4. doi: 10.1007/s10577-017-9551-2 (PMC5346143; doi:10.1007/s10577-017-9551-2)

# A

## Contact maps (chr 19)

Simulations

Hi-C (Rao et al.)

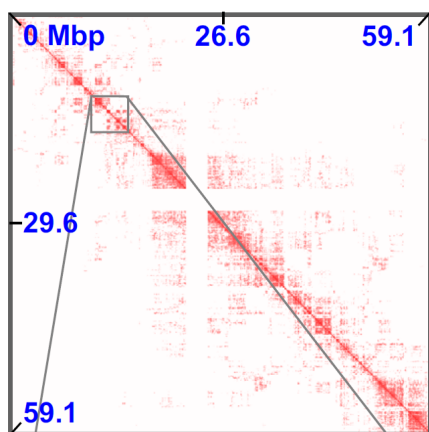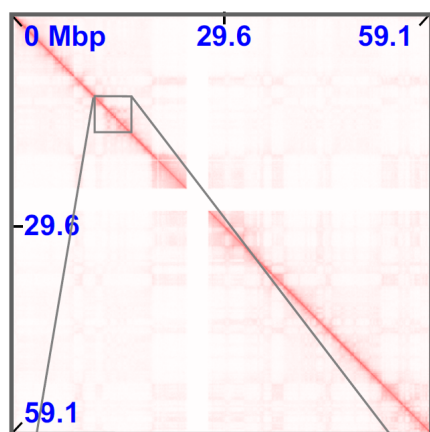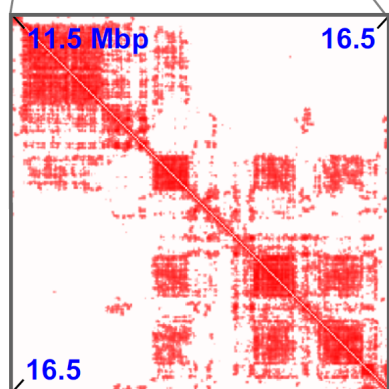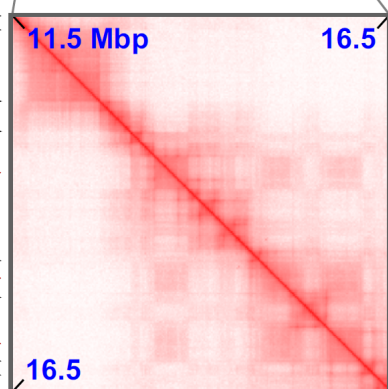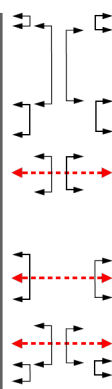

# B

## Simulation snapshots (chr 19)

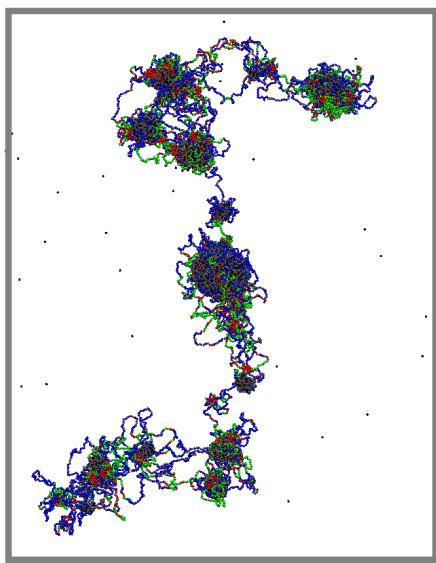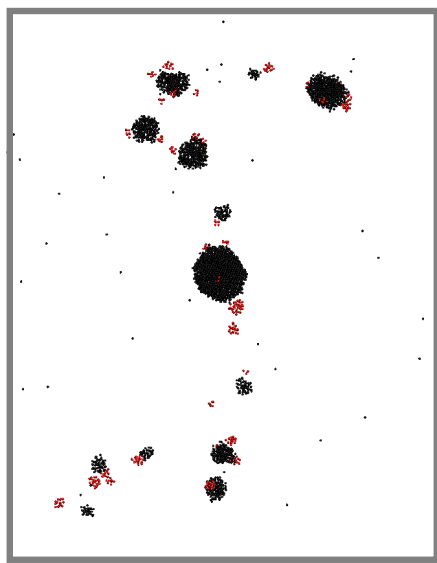

Supplement: Supplementary file 1 — (PDF 1137 kb) [file 10577_2017_9551_MOESM1_ESM.pdf]
